# Correction to: MicroRNA-214-3p inhibits proliferation and cell cycle progression by targeting MELK in hepatocellular carcinoma and correlates cancer prognosis

**DOI:** 10.1186/s12935-018-0556-5

**Published:** 2018-04-10

**Authors:** Yue Li, You Li, Yao Chen, Qian Xie, Ningning Dong, Yanjun Gao, Huan Deng, Chunhua Lu, Suihai Wang

**Affiliations:** 1 Department of Laboratory Medicine, Nanfang Hospital, Southern Medical University, Guangzhou, 510515 Guangdong Province China; 2 College of Life Science and Technology, Guangxi University, Nanning, 530004 Guangxi Province China; 3 Institute of Antibody Engineering, School of Laboratory Medicine and Biotechnology, Southern Medical University, Guangzhou, 510515 Guangdong Province China

## Correction to: Cancer Cell Int (2017) 17:102 10.1186/s12935-017-0471-1

After publication of the original article [1] it was noted that the author details and affiliations of this manuscript contained minor errors in the institution addresses.

The correct affiliations for each of the authors are listed below:

Yue Li—Department of Laboratory Medicine, Nanfang Hospital, Southern Medical University, Guangzhou 510515, Guangdong Province, China.

You Li—College of Life Science and Technology, Guangxi University, Nanning 530004, Guangxi Province, China.

Yao Chen—Institute of Antibody Engineering, School of Laboratory Medicine and Biotechnology, Southern Medical University, Guangzhou 510515, Guangdong Province, China.

Qian Xie—Institute of Antibody Engineering, School of Laboratory Medicine and Biotechnology, Southern Medical University, Guangzhou 510515, Guangdong Province, China.

Ningning Dong—Institute of Antibody Engineering, School of Laboratory Medicine and Biotechnology, Southern Medical University, Guangzhou 510515, Guangdong Province, China.

Yanjun Gao—Institute of Antibody Engineering, School of Laboratory Medicine and Biotechnology, Southern Medical University, Guangzhou 510515, Guangdong Province, China.

Huan Deng—Institute of Antibody Engineering, School of Laboratory Medicine and Biotechnology, Southern Medical University, Guangzhou 510515, Guangdong Province, China.

Chunhua Lu*—College of Life Science and Technology, Guangxi University, Nanning 530004, Guangxi Province, China.

Suihai Wang*—Institute of Antibody Engineering, School of Laboratory Medicine and Biotechnology, Southern Medical University, Guangzhou 510515, Guangdong Province, China.
